# Description of the genetic variants identified in a cohort of patients diagnosed with localized anal squamous cell carcinoma and treated with panitumumab

**DOI:** 10.1038/s41598-021-86966-w

**Published:** 2021-04-01

**Authors:** Lucía Trilla-Fuertes, Angelo Gámez-Pozo, Joan Maurel, Rocio Garcia-Carbonero, Jaume Capdevila, Laura G-Pastrián, Marta Mendiola, Cristina Peña, Rocío López-Vacas, Miriam Cuatrecasas, Pilar García-Alfonso, Ricardo Ramos-Ruiz, Carlos Llorens, Ismael Ghanem, Carles Conill, Victoria Heredia-Soto, Ángel Campos-Barros, Juan Ángel Fresno Vara, Jaime Feliu

**Affiliations:** 1 Biomedica Molecular Medicine SL, Madrid, Spain; 2 Molecular Oncology and Pathology Lab, Hospital Universitario La Paz-IdiPAZ, Madrid, Spain; 3 Medical Oncology Department, Hospital Clinic of Barcelona, Translational Genomics and Targeted Therapeutics in Solid Tumors Group, IDIBAPS, University of Barcelona, Barcelona, Spain; 4 Medical Oncology Department, Instituto de Investigación Sanitaria Hospital 12 de Octubre (imas12), UCM, CNIO, CIBERONC, Hospital Universitario 12 de Octubre, Madrid, Spain; 5 Medical Oncology Service, Vall Hebron Institute of Oncology (VHIO), Vall Hebron University Hospital, Barcelona, Spain; 6 Pathology Department, Hospital Universitario La Paz, Madrid, Spain; 7 Molecular Pathology and Therapeutic Targets Group, Hospital Universitario La Paz-IdiPAZ, Madrid, Spain; 8 Biomedical Research Networking Center On Oncology-CIBERONC, ISCIII, Madrid, Spain; 9 Pathology Department, Hospital Clínic Universitat de Barcelona, Villarroel 170, 08036 Barcelona, Spain; 10 Medical Oncology Department, Hospital General Universitario Gregorio Marañón, Madrid, Spain; 11 Genomics Unit Cantoblanco, Parque Científico de Madrid, C/ Faraday 7, 28049 Madrid, Spain; 12 Biotechvana SL, Parque Científico de Madrid, C/ Faraday 7, 28049 Madrid, Spain; 13 Medical Oncology Department, Hospital Universitario La Paz, Paseo de la Castellana 261, 28046 Madrid, Spain; 14 Radiotherapy Oncology Department, Hospital Clinic of Barcelona, Carrer de Villarroel 170, 08036 Barcelona, Spain; 15 Translational Oncology Group, Hospital Universitario La Paz-IdiPAZ, Madrid, Spain; 16 Institute of Medical and Molecular Genetics, IdiPAZ, Unit 753, ISCIII, Hospital Universitario La Paz /& CIBERER, Paseo de la Castellana 261, 28046 Madrid, Spain; 17 Cátedra UAM-Amgen, Universidad Autónoma de Madrid, Madrid, Spain

**Keywords:** Cancer, Medical genetics, Predictive markers

## Abstract

Squamous cell carcinoma is the most frequent histologic type of anal carcinoma. The standard of care since the 1970s has been a combination of 5-fluorouracil, mitomycin C, and radiotherapy. This treatment is very effective in T1/T2 tumors (achieving complete regression in 80–90% of tumors). However, in T3/T4 tumors, the 3-year relapse free survival rate is only 50%. The VITAL trial aimed to assess the efficacy and safety of panitumumab in combination with this standard treatment. In this study, 27 paraffin-embedded samples from the VITAL trial and 18 samples from patients from daily clinical practice were analyzed by whole-exome sequencing and the influence of the presence of genetic variants in the response to panitumumab was studied. Having a moderate- or high-impact genetic variant in *PIK3CA* seemed to be related to the response to panitumumab. Furthermore, copy number variants in *FGFR3*, *GRB2* and *JAK1* were also related to the response to panitumumab. These genetic alterations have also been studied in the cohort of patients from daily clinical practice (not treated with panitumumab) and they did not have a predictive value. Therefore, in this study, a collection of genetic alterations related to the response with panitumumab was described. These results could be useful for patient stratification in new anti-EGFR clinical trials.

## Introduction

Anal squamous cell carcinoma (ASCC) is the most frequent histologic type of anal carcinoma. An estimated 8300 new diagnoses were predicted in the United States in 2019, representing 2.5% of gastrointestinal cancers^1^. The combination of 5-fluorouracil (5-FU), mitomycin C (MMC), and radiotherapy (RT) has been established as the standard of care in Europe and the U.S. since the 1970s^2,3^. This treatment achieves a 5-year overall survival (OS) rate of 70–80%^4,5^ and is particularly very effective in cT1/T2 tumors, achieving complete regression in 80%-90% of cases, permanently in most of them. However, in large anal tumors (T3-4 or N +), the 3-year disease-free survival (DFS) rate is around 50%^6^.

The incidence of ASCC has been associated with many risk factors, most importantly with human papillomavirus (HPV) infection^7^. The HPV-associated E5 protein amplifies the mitogenic signals through the epidermal growth factor receptor (EGFR) pathway^8^ which is broadly expressed in squamous cell carcinomas of the anogenital tract and oropharynx^9–11^.

EGFR activation causes multiple effects, including cell proliferation, migration, adhesion, angiogenesis, and inhibition of apoptosis^12,13^. Monoclonal EGFR antibodies prevent the activation of signaling transduction pathways mediated by EGFR. Panitumumab and cetuximab are the two EGFR antibodies most used in clinical practice. They bind to the extracellular domain of the EGFR monomer and compete for receptor binding by the endogenous ligands, blocking ligand-induced receptor activation^14^. EGFR is highly expressed in ASCC^15^ and several clinical trials using an anti-EGFR therapy exist. However, despite the molecular evidence for the use of anti-EGFR therapy, these trials have not demonstrated its efficacy in the population with ASCC^16–18^. The VITAL study (GEMCAD-09-02, NCT01285778), a phase II multicenter, single-arm study, aimed to assess the efficacy and safety of panitumumab in combination with the standard treatment of localized ASCC tumors (5-fluorouracil (5FU), mitomycin C and radiotherapy). However, the conclusion of this trial was that the panitumumab addition to standard chemotherapy increased toxicity and did not improve patients’ outcomes^19^. In this work, we have studied samples from this clinical trial and from a cohort treated only with chemoradiotherapy to define possible mechanisms of resistance and to suggest how to select patients in future studies.

In addition, the mutational status of certain genes has demonstrated its utility as a predictor of response to treatment in other cancers. That is the case, for instance, with KRAS status, which is a strong biomarker of negative response to EGFR antibodies in colorectal cancer patients^20–22^.

The objective of this study was to describe the genetic variants that could be associated with presentation of a negative response to panitumumab and that could be used to stratify the ASCC population in future clinical trials.

## Results

### Patient cohort

Twenty-seven patients from the VITAL clinical trial (GEMCAD-09–02, NCT01285778) were studied. These patients received panitumumab, 5FU and mitomycin C concomitantly with radiotherapy. The endpoint of this clinical trial was disease-free survival (DFS). Eight patients (30%) out of 27 had a relapse event. At 24 months, the DFS rate was 70%.

In order to compare patients treated with chemoradiotherapy and panitumumab with those treated only with chemoradiotherapy, a cohort of eighteen patients from daily clinical practice at Hospital Universitario La Paz and the Hospital Clinic of Barcelona was used. Of these eighteen patients, eight patients presented a relapse (44%). At 24 months, the DFS rate was 54%. All patient characteristics are shown in Table 1.

**Table 1:** Patients’ characteristics.

	VITAL cohort	Chemo-only cohort
Number of patients	27	18
Age at diagnosis (median and range)	63 (42–83)	59 (41–86)
Age at diagnosis (mean)	62	59
**Gender**		
Male	13 (48%)	10 (56%)
Female	14 (52%)	8 (44%)
**HPV**		
16	17 (63%)	9 (50%)
Other subtypes	5 (19%)	3 (16.3%)
Negative	2 (7%)	3 (16.3%)
Unknown	3 (11%)	3 (16.3%)
**HIV**		
Positive	0 (0%)	2 (11%)
Negative	27 (100%)	16 (89%)
**T**		
< 5 cm	16 (59%)	12 (66%)
> 5 cm	9 (33%)	3 (17%)
Unknown	2 (8%)	3 (17%)
**Lymph node status**		
N0	8 (30%)	11 (61%)
N positive	17 (63%)	7 (39%)
Unknown	2 (7%)	0 (0%)
**TNM stage**		
I	0 (0%)	3 (16.3%)
II	8 (30%)	8 (44.7%)
III	19 (70%)	7 (39%)

**Figure 1:**
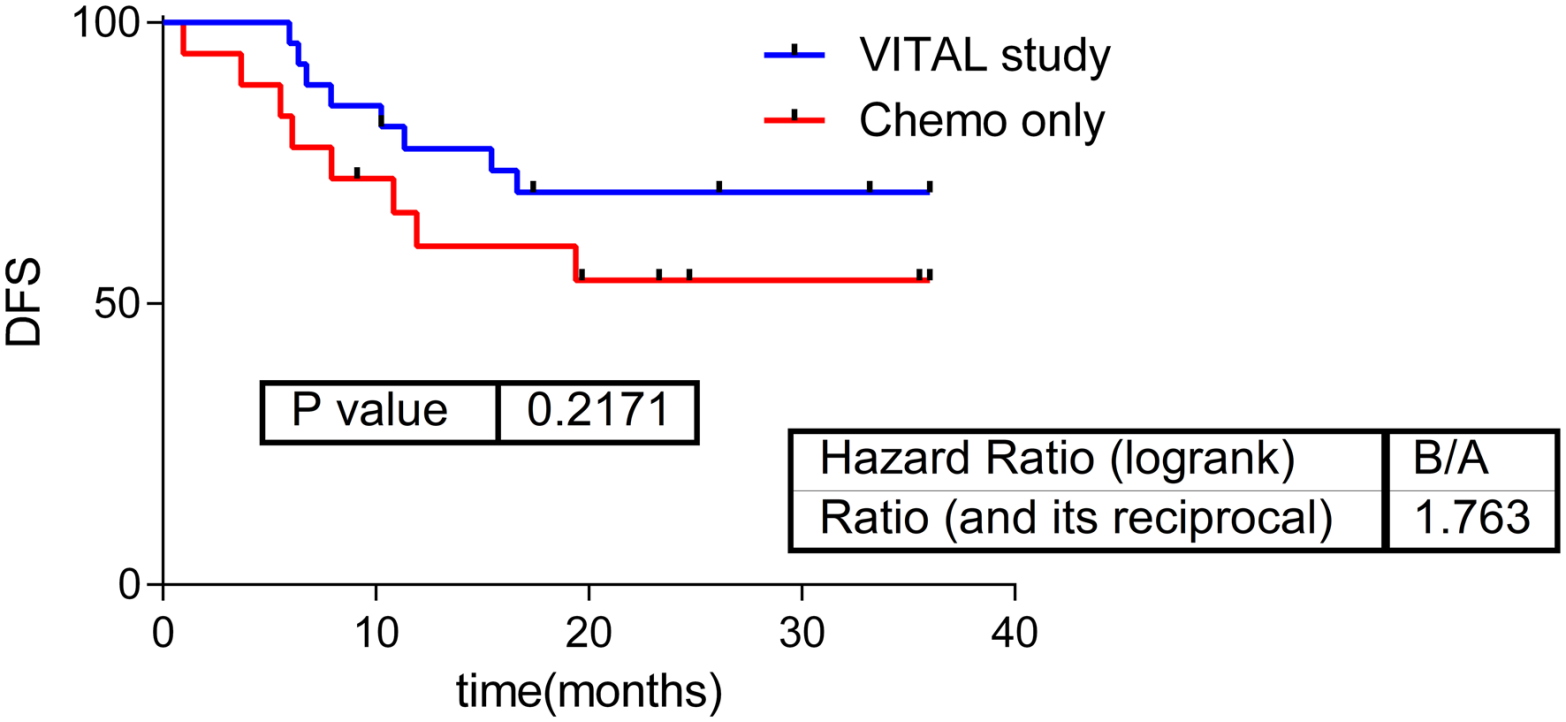
Survival curves. Survival curves in patients from the VITAL clinical trial treated with chemotherapy, radiotherapy, and panitumumab; and patients from daily clinical practice (treated only with chemotherapy plus radiotherapy). *DFS* Disease-free survival.

There were no significant differences in DFS between patients receiving chemoradiotherapy with and without panitumumab (Fig. 1).

(*p* = 0.217, HR = 1.76).

### Quality of whole-exome sequencing experiments

Mean coverage shown in WES experiments was > 42.6x. The mapping efficiency was 90–98%, with the exception of one sample (75.4%). The human exome contains > 195,000 exonic regions, of which only 23,021 (11.21%) were not mapped in any sample.

### Genetic variants identified in genes involved in the panitumumab pharmacodynamics pathway

The EGFR inhibitor pharmacodynamics pathway was obtained from the PharmGKB database (https://www.pharmgkb.org/). We studied the presence of genetic variants and CNVs in genes of this pathway in the eight patients that did not respond to treatment (i.e. had a relapse).

Of these eight patients, four of them presented a high- or moderate-impact genetic variant in the *PIK3CA* gene. The genetic variants were chr3_178917478_G/A (rs587777790) and chr3_178936091_G/A (rs104886003) (shown in two patients). The rs587777790 variant is a missense substitution and causes Gly110 to change to Asp. It is classified by ClinVar (https://www.ncbi.nlm.nih.gov/clinvar/) as pathogenic in Cowden syndrome and as likely to be pathogenic in several neoplasms. The rs104886003 variant is a missense substitution that causes Glu545 to change to Lys. It is classified by ClinVar as pathogenic in neoplasms of the large intestine.

Another patient presented a deletion of *PIK3CA* (chr3_178937459_T/-), which creates a frameshift starting at codon Arg617. The new reading frame ends in a STOP codon at position 7. This deletion was classified by ClinVar as pathogenic in the colon and neoplasms of the large intestine.

In addition, four patients presented a duplication of the *GRB2* gene and two carried a duplication of the *JAK1* gene, both of which are also involved in the pharmacodynamics pathway of EGFR inhibitors. Two other patients had a duplication of the *EGFR* and one patient presented a deletion of this gene but these were not related to DFS.

**Figure 2:**
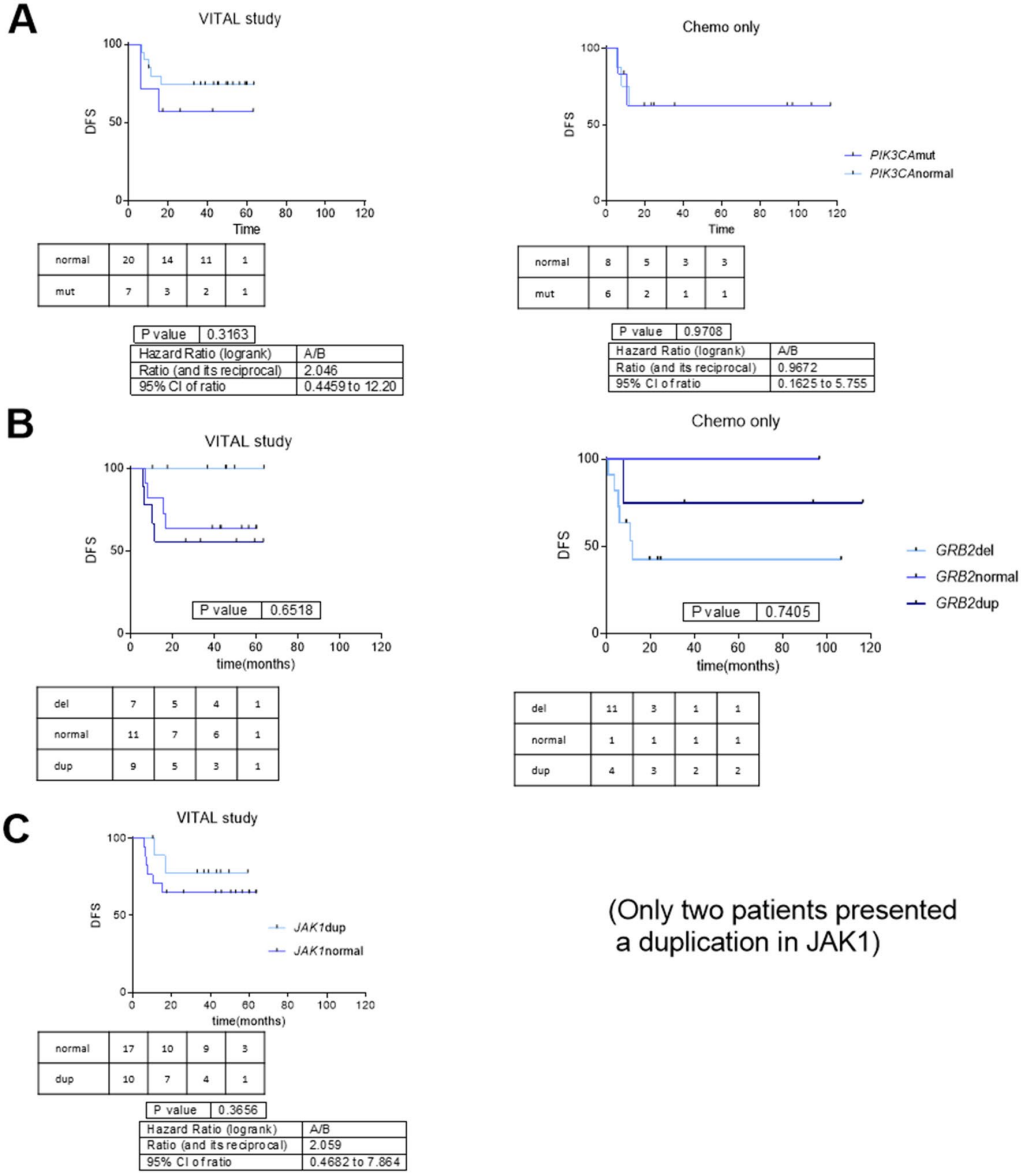
Disease-free survival curves for relevant genetic variants in pharmacodynamics pathway of EGFR inhibitors. (**A**) Disease-free survival curves for patients with high- and moderate-impact genetic variants in *PIK3CA* versus *PIK3CA* wild-type in patients from the VITAL clinical trial and from the chemoradiotherapy cohort. (**B**) Disease-free survival curves for VITAL clinical trial and chemoradiotherapy cohort patients according to *GRB2* status. HR between normal-duplicated vs deletion = 4.19 (CI95% 0.92–19.2). (**C**) Disease-free survival curves for VITAL clinical trial patients according to *JAK1* status. *DFS* Disease-free survival.

Regarding DFS, no alteration in any gene presented statistically significant differences. However, hazard ratios (HR) were all above 2 in the case of *PIK3CA*, *GRB2* and *JAK1*. In the cohort of patients from daily clinical practice these genes did not seem to have a relationship with DFS (Fig. 2).

### Genetic variants in genes that had been previously been related to EGFR inhibitor response

In previous works, *BRAF* inactivation or *PTEN* deletion had also been associated with a worse response to EGFR inhibitors^23,24^. For this reason, we characterized the alterations presented in these genes in our cohort. Two patients out of eight that did not respond to panitumumab presented a *PTEN* gene deletion.

Furthermore, another of these eight patients had a point mutation in *BRAF*. Alamut splicing predictors classified this variant as a splice site substitution located in the acceptor splice site of intron 13. The consequence of this change is not predictable but a skip of exon 14 is very likely.

All genetic variants shown in these genes are summarized in Table 2.

**Table 2:** Genetic variants in genes involved in the pharmacodynamics pathway of EGFR inhibitors and genetic variants in *PTEN* and *BRAF*, genes previously described in the literature as being related to the response to EGFR inhibitors.

Pts who relapsed	*PIK3CA* mut	*PTEN* del	*BRAF* mut	*GRB2* dup	*JAK1* dup	*EGFR* dup	*EGFR* del
CAN23		chr10:89472580–90356944					
CAN29	chr3_178917478_G/A NM_006218.2:c.353G > A p.(Gly118Asp)			chr17:72860820–73759748			
CAN34	chr3_178937459_T/-NM_006218.2:c.1848del p.(Arg617Glyfs*7)					chr7:54612063–55565645	
CAN38	chr3_178936091_G/A NM_006218.2:c.1633G > A p.(Glu545Lys)			chr17:73126331–73539845			
CAN50					chr1:62236851–65830719		chr7:47860422–56067033
CAN52	chr3_178936091_G/A NM_006218.2:c.1633G > A p.(Glu545Lys)			chr17:72968417–73759748	chr1:64017144–67862023		
CAN55		chr10:75531840–91465449	chr7_140454035_T/A NM_001354609.1:c.1695-2A > T Not predictable	chr17:48046581–73539845			
CAN67						chr7:44146772–57189110	

**Figure 3:**
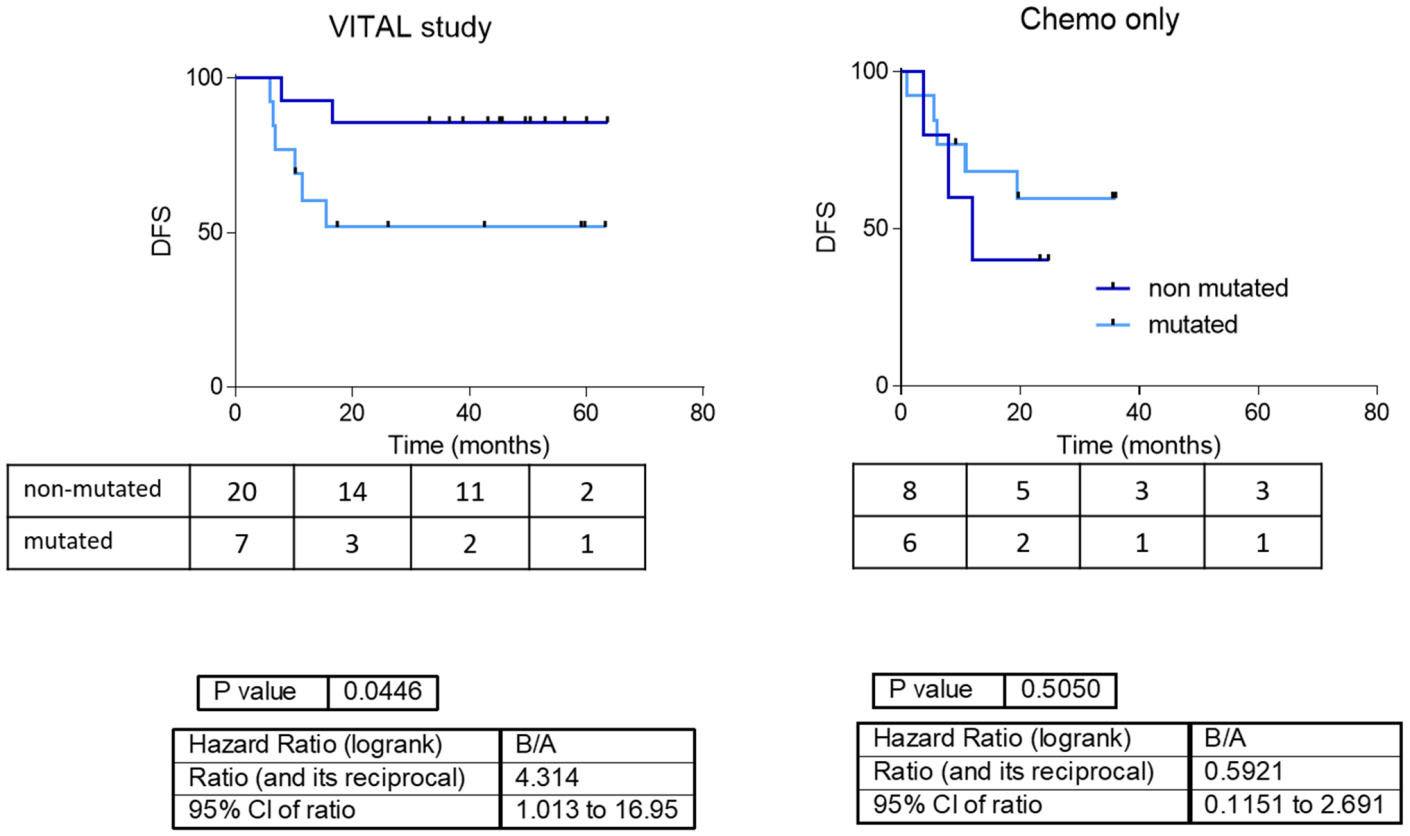
Survival curves according mutation accumulation in pharmacodynamics pathway of EGFR inhibitors. Survival curves grouping patients from the VITAL clinical trial and from the chemoradiotherapy cohort according to the presence of high- and moderate-impact genetic variants in genes involved in the pharmacodynamics pathway of EGFR inhibitors.

Finally, the high- and moderate-impact variants in genes included in the EGFR inhibitor pharmacodynamics pathway seems to be related to DFS when grouped together. Patients without any variation in those genes had a better prognosis than patients with one or more genetic variants (Fig. 3). The accumulation of genetic variants in the genes of the EGFR inhibitor pharmacodynamics pathway did not split the chemotherapy-only population according to DFS.

### Genetic variants in EGFR family receptors

**Figure 4:**
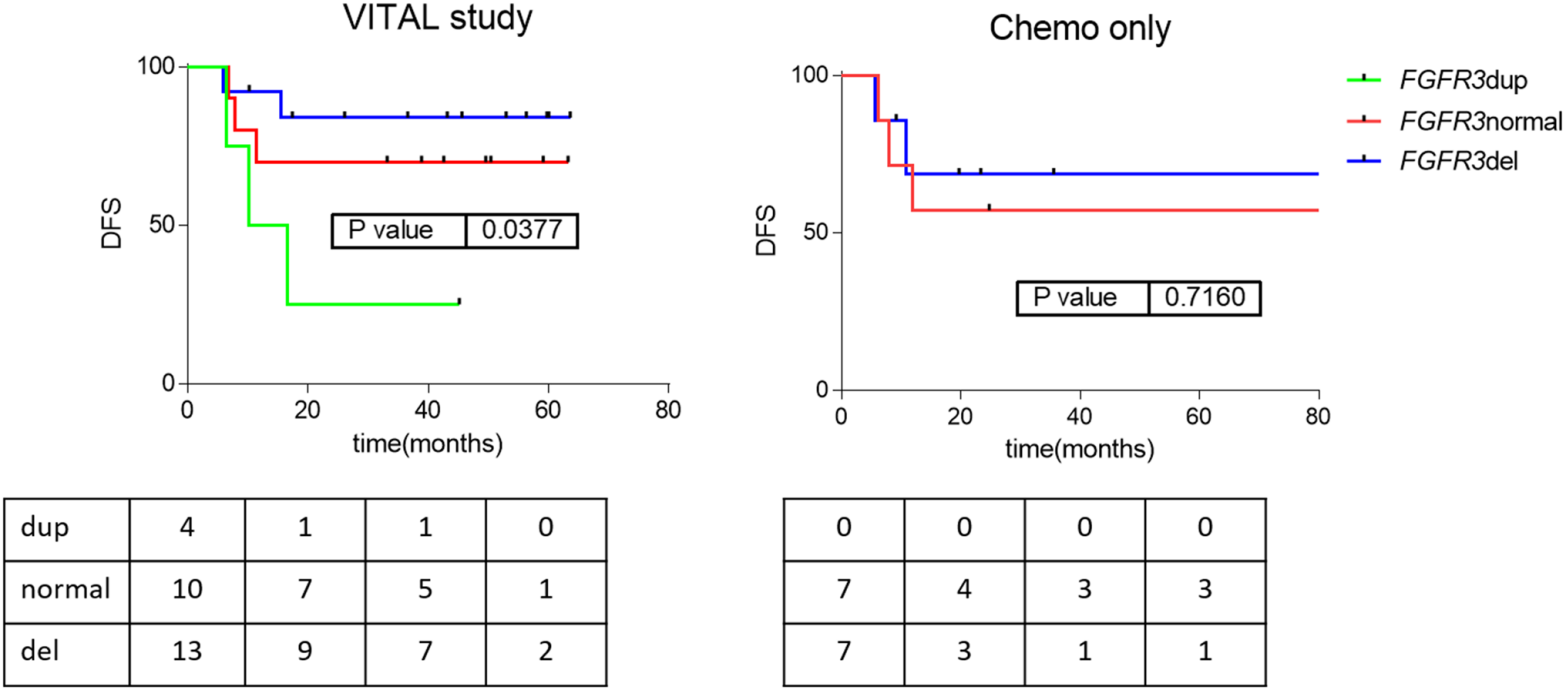
Survival curves for relevant EGFR-family receptors. Disease-free survival curves for VITAL clinical trial and chemoradiotherapy cohort patients according to *FGFR3* status. HR between normal-del vs duplication = 4.30 (CI95% 1.28–88.25) dup: *FGFR3* duplication; normal: *FGFR3* wild type; del: *FGFR3* deletion.

Studying the EGFR pathway in KEGG (https://www.genome.jp/kegg/pathway.html), it is shown that several receptors are similar to EGFR: FGFR3, ERBB2, ERBB3, and IGF1R. We studied if the presence of genetic alterations or CNVs in these receptors were related to the response to panitumumab. Strikingly, the number of copies of the *FGFR3* gene seems to be related to disease-free survival in VITAL patients. Again, in the cohort of patients from daily clinical practice these genes did not seem to have a relationship with DFS (Fig. 4). This suggests that these genes were related to the response to panitumumab rather than to the prognosis.

## Discussion

ASCC is a rare tumor and its treatment has not been improved since the 1970s. Although ASCC tumors overexpress EGFR, the results of clinical trials with EGFR antibodies were negative, even several trials showed more toxicity but not more efficacy^16–18^. In this study, we characterized WES genetic alterations that could be related to resistance to panitumumab treatment.

Firstly, genes defined in PharmGKB as being involved in the EGFR inhibitor pharmacodynamics pathway were studied^14^. Four patients that relapsed presented a genetic variant in *PIK3CA*. *PIK3CA* mutations in colorectal cancer have been previously associated with resistance to EGFR inhibitors^25^. Two patients that relapsed had a deletion of the *PTEN* gene. *PTEN* inactivation was defined as a negative predictor of response to EGFR inhibitors in colorectal cancer^23,24^. Finally, one patient showed a mutation in *BRAF*, in the splice acceptor site of intron 13. The consequence of this change is not predictable by Alamut pathogenic predictors but a skip of exon 14 is very likely. Several studies have previously identified that BRAF V600E, which is a missense substitution located in exon 15 and causes *BRAF* constitutive activation, is associated with a poor prognosis in colorectal cancer^24^. Additionally, it has been established that the levels of EGFR expression were not correlated with the clinical response to cetuximab or panitumumab in colorectal cancer patients^24^. We did not show any correlation between the number of copies of *EGFR* and DFS in our ASCC cohort either.

Secondly, similar receptors to EGFR were studied. The number of copies of *FGFR3* seems to be related to DFS in ASCC patients treated with panitumumab. Oliveras-Ferraros et al. described FGFR3 hyperactivation in cetuximab-resistant epidermoid carcinoma cells and identified a synergistic effect in the combination of cetuximab plus an FGFR3 inhibitor in these cells^26^. In addition, Ware et al. associated an acquisition of resistance to EGFR inhibitors in non-small cell lung cancer with FGFR3 and FGFR2 activation^27^. These results agreed with the lower DFS shown in ASCC patients with a *FGFR3* duplication treated with panitumumab.

It is remarkable that these genetic variants seem not to have a relationship with prognosis in the cohort of patients treated only with chemoradiotherapy and not with panitumumab. This suggests that these alterations were probably not related to prognostic prediction, since in that case these variants would also have prognostic value in the cohort of patients not treated with panitumumab, although they were related to a lack of response to panitumumab treatment.

These results could be useful in other diseases where anti-EGFR treatment is proposed. For instance, cetuximab and panitumumab showed debatable results in head and neck squamous cell carcinoma^28^.The presence of a biomarker could therefore help to guide anti-EGFR treatment in these tumors.

The main limitation of the study is that an independent validation of the relationship between these genetic variants and the response to panitumumab in ASCC is needed, followed by functional validation of validated moderate variants. Another limitation is the small number of samples due to the fact that ASCC is a rare tumor and available information is scarce.

In this study, a collection of genetic variants and genes that were related to the response to panitumumab in ASCC were described. Clinical trials evaluating anti-EGFR efficacy in a localized ASCC population have recently failed. Molecular biomarkers, including mutations, have demonstrated their utility in predicting responses to drugs, so it seems useful to identify genetic alterations in ASCC patients that had a poor response to anti-EGFR therapy. Additionally, these results could help in the design of new anti-EGFR therapy studies. Finally, the results of this study suggest the potential usefulness of molecular stratification of patients in clinical trials.

## Methods

### Patient cohort

Twenty-seven patients recruited from the VITAL clinical trial (GEMCAD-09–02, NCT01285778) were included in this study. These patients had received 5FU and mitomycin C concomitantly with radiotherapy, and panitumumab. The recruitment period was from October 2010 to March 2017. Patients received treatment with panitumumab (Vectibix; Amgen) 6 mg/kg intravenously (IV) on day 1 and every 2 weeks for 8 weeks. Panitumumab treatment was followed by 5-FU 1000 mg/m2 /d by continuous IV infusion on days 1–4 and 29–32, and Mitomycin C 10 mg/m2 IV on days 1 and 29. RT was given on days 1–37 to a total dose of 45 Gy (1.8 Gy/fraction, 5 fractions per week) to the primary tumor and mesorectal, iliac and inguinal lymph nodes, plus a boost dose of 10–15 Gy to the primary tumor and affected lymph nodes. Intensity modulated radiation therapy or 3-D conformal radiotherapy was used depending on the center’s availability following protocol guidelines.

Eighteen patients from daily clinical practice in Hospital Universitario La Paz and the Hospital Clinic of Barcelona were included as a control group. These patients had received chemotherapy (5FU and mitomycin C) concomitantly with radiotherapy, but they were not treated with panitumumab or another EGFR inhibitor. The recruitment period was from January 1st 2006 to December 30th 2016.

Inclusion criteria included patients older than 18 years old, with histologically confirmed ASCC, with an Eastern Cooperative Oncology Group performance status (ECOG-PS) of 0 to 2, without previous radiotherapy or chemotherapy, and without distant metastasis. Demographic variants, related to both tumor and treatments, were collected. Human papillomavirus (HPV) infection was determined using CLART HPV2 (Genomica). All patients are from white ethnicity. This study was approved by Hospital Universitario La Paz Ethics Committee. Informed consent was obtained for all the patients enrolled in the study. Disease-free survival (DFS) is defined as the time since the first dose of treatment to date of the first treatment failure, defined as local, regional or distant failure, second primary tumor, or death for every cause. Overall survival (OS) is defined as the time since the first dose of treatment to date of death.

### Sample processing and DNA isolation

Tumor samples without any prior treatment were obtained and reviewed by a pathologist and only those with 70% tumor cells were selected. One 10-mm section from each FFPE sample was deparaffinized and DNA was extracted using the GeneRead DNA FFPE Kit (Qiagen), following the manufacturer’s instructions. Once eluted, DNA was frozen at -80 °C until use.

### Whole-exome sequencing experiments

Whole-exome sequencing (WES) from 45 FFPE samples of ASCC (27 from the VITAL trial and 18 from daily clinical practice) was performed. Purified DNA was quantified by Picogreen and mean size was determined by gel electrophoresis. Genomic DNA was fragmented by mechanical means (Bioruptor) to a mean size of around 200 bp. Then, DNA samples were repaired, phosphorylated, A-tailed and ligated to specific adaptors, followed by PCR-mediated labeling with Illumina-specific sequences and sample-specific barcodes (Kapa DNA library generation kit).

Exome capture was performed using the VCRome system (capture size of 37 Mb, hg19 was used as reference, Roche Nimblegen) with multiplexing of 8 samples per capture reaction. Capture was carried out with strict adherence to the manufacturer’s instructions. After capture, libraries were purified, quantified and titrated by Real Time PCR before sequencing. Samples were then sequenced to an approximate coverage of 4.5 Gb per sample in Illumina-NextSeq NS500 (Illumina Inc.) using 150-cycle (2 × 75) High Output cartridges.

### Bioinformatics analyses

The quality of the WES experiments was checked using FASTQC (http://www.bioinformmatics.babraham.ac.uk/projects/fastqc). First, adaptors were removed using Cutadapt^29^. Then, FASTQ files were filtered by quality using PrinSeq^30^. Both tools are included in the GPRO Suite (Biotechvana)^31^. Sequences were then aligned using the human genome h19 as a reference genome, using the BWA^32^, Samtools^33^ and Picardtools (http://picard.sourceforge.net) tools. For variant calling, the MuTect tool from the GATK4 package^34^, combined with PicardTools were used, firstly to create a panel of normal samples (PON) using 11 samples from Iberian exomes from 1000 genomes (http://www.ncbi.nlm.nih.gov/sra/) to discard germline variants and, secondly, for the variant calling^35^.

Copy number variants (CNVs) were called using the GATK4 package^34^ and PON to standardize copy proportions and remove noise from the counting data of each sample.

### Interpretation of the genetic variants

The PharmGKB database (https://www.pharmgkb.org/) and KEGG (https://www.genome.jp/kegg/pathway.html) were used to select genes included in the EGFR inhibitor pharmacodynamics pathway and in the EGFR pathway respectively. The effect and pathogenicity of each genetic variant were assessed using Alamut Visual v2.11 (Interactive Biosoftware). Moderate (missense) and high impact genetic variants (frameshift, splice variants, stop-loss and stop-gain variants), are defined according VEP criteria of the impact of each mutation in the corresponding gene.

### Statistical analyses

Statistical analyses were performed in GraphPad Prism v6. Kaplan–Meier and log-rank tests were used for survival analysis. *p* values were two-sided and considered statistically significant under 0.05.

### Ethical statement

All research was performed in accordance with relevant guidelines/regulations. Written consent was obtained from all patients. This study was approved by Hospital Universitario La Paz Ethics Committee (PI-1926).

## Data Availability

Whole-exome sequencing raw data files are available in Sequence Read Archive (SRA, https://www.ncbi.nlm.nih.gov/sra) under the name PRJNA573670.

## References

[1] Siegel RL, Miller KD, Jemal A (2019). Cancer statistics, 2019. CA Cancer J. Clin..

[2] Ryan DP, Compton CC, Mayer RJ (2000). Carcinoma of the anal canal. N. Engl. J. Med..

[3] Doci R (1996). Primary chemoradiation therapy with fluorouracil and cisplatin for cancer of the anus: results in 35 consecutive patients. J. Clin. Oncol..

[4] Gerard JP (1998). Treatment of anal canal carcinoma with high dose radiation therapy and concomitant fluorouracil-cisplatinum. Long-term results in 95 patients. Radiother. Oncol..

[5] Allal A (1993). Chemoradiotherapy versus radiotherapy alone for anal cancer: a retrospective comparison. Int. J. Radiat. Oncol. Biol. Phys..

[6] Uronis HE, Bendell JC (2007). Anal cancer: an overview. Oncologist.

[7] Morton M, Melnitchouk N, Bleday R (2018). Squamous cell carcinoma of the anal canal. Curr. Probl. Cancer.

[8] Crusius K, Auvinen E, Alonso A (1997). Enhancement of EGF- and PMA-mediated MAP kinase activation in cells expressing the human papillomavirus type 16 E5 protein. Oncogene.

[9] Taberna M (2018). The use of HPV16-E5, EGFR, and pEGFR as prognostic biomarkers for oropharyngeal cancer patients. Front. Oncol..

[10] Wechsler EI, Tugizov S, Herrera R, Da Costa M, Palefsky JM (2018). E5 can be expressed in anal cancer and leads to epidermal growth factor receptor-induced invasion in a human papillomavirus 16-transformed anal epithelial cell line. J. Gen. Virol..

[11] Maufort JP, Shai A, Pitot HC, Lambert PF (2010). A role for HPV16 E5 in cervical carcinogenesis. Cancer Res..

[12] Arteaga CL (2001). The epidermal growth factor receptor: from mutant oncogene in nonhuman cancers to therapeutic target in human neoplasia. J. Clin. Oncol..

[13] Baselga J (2002). Why the epidermal growth factor receptor? The rationale for cancer therapy. Oncologist.

[14] Whirl-Carrillo M (2012). Pharmacogenomics knowledge for personalized medicine. Clin. Pharmacol. Ther..

[15] Alvarez G, Perry A, Tan BR, Wang HL (2006). Expression of epidermal growth factor receptor in squamous cell carcinomas of the anal canal is independent of gene amplification. Mod. Pathol..

[16] Casadei Gardini A (2018). Treatment of squamous cell carcinoma of the anal canal: a new strategies with anti-EGFR therapy and immunotherapy. Crit. Rev. Oncol. Hematol..

[17] Garg MK (2017). Cetuximab plus chemoradiotherapy in immunocompetent patients with anal carcinoma: a phase II Eastern Cooperative Oncology Group-American College of Radiology Imaging Network Cancer Research Group Trial (E3205). J. Clin. Oncol..

[18] Sparano JA (2017). Cetuximab plus chemoradiotherapy for HIV-associated anal carcinoma: a phase II AIDS malignancy consortium trial. J. Clin. Oncol..

[19] Feliu J (2020). VITAL phase 2 study: upfront 5-fluorouracil, mitomycin-C, panitumumab and radiotherapy treatment in nonmetastatic squamous cell carcinomas of the anal canal (GEMCAD 09–02). Cancer Med..

[20] Custodio A, Feliu J (2013). Prognostic and predictive biomarkers for epidermal growth factor receptor-targeted therapy in colorectal cancer: beyond KRAS mutations. Crit. Rev. Oncol. Hematol..

[21] De Roock W (2008). KRAS wild-type state predicts survival and is associated to early radiological response in metastatic colorectal cancer treated with cetuximab. Ann. Oncol..

[22] Okada Y, Miyamoto H, Goji T, Takayama T (2014). Biomarkers for predicting the efficacy of anti-epidermal growth factor receptor antibody in the treatment of colorectal cancer. Digestion.

[23] Therkildsen C, Bergmann TK, Henrichsen-Schnack T, Ladelund S, Nilbert M (2014). The predictive value of KRAS, NRAS, BRAF, PIK3CA and PTEN for anti-EGFR treatment in metastatic colorectal cancer: a systematic review and meta-analysis. Acta Oncol..

[24] Bardelli A, Siena S (2010). Molecular mechanisms of resistance to cetuximab and panitumumab in colorectal cancer. J. Clin. Oncol..

[25] Sartore-Bianchi A (2009). PIK3CA mutations in colorectal cancer are associated with clinical resistance to EGFR-targeted monoclonal antibodies. Cancer Res..

[26] Oliveras-Ferraros C (2012). Cross-suppression of EGFR ligands amphiregulin and epiregulin and de-repression of FGFR3 signalling contribute to cetuximab resistance in wild-type KRAS tumour cells. Br. J. Cancer.

[27] Ware KE (2010). Rapidly acquired resistance to EGFR tyrosine kinase inhibitors in NSCLC cell lines through de-repression of FGFR2 and FGFR3 expression. PLoS One.

[28] Oliveira-Silva RJ, Carolina de Carvalho A, deSouzaViana L, Carvalho AL, Reis RM (2016). Anti-EGFR therapy: strategies in head and neck squamous cell carcinoma. Recent Pat. Anticancer Drug Discov..

[29] Martin M (2011). Cutadapt removes adapter sequences from high-throughput sequencing reads. EMBnet.journal.

[30] Schmieder R, Edwards R (2011). Quality control and preprocessing of metagenomic datasets. Bioinformatics.

[31] Futami, R. *et al.* GPRO The professional tool for annotation, management and functional analysis of omic databases. *Biotechvana Bioinform. SOFT3*. https://gpro.biotechvana.com/citing (2011).

[32] Li, H. & Durbin, R. Fast and accurate short read alignment with Burrows-Wheeler transform. *Bioinformatics* **25(14)**, 1754–1760, 10.1093/bioinformatics/btp324. Epub 2009 May 18. (2009). 10.1093/bioinformatics/btp324 PMC2705234 19451168

[33] Li H (2009). The sequence alignment/map format and SAMtools. Bioinformatics.

[34] McKenna, A. *et al.* The Genome Analysis Toolkit: a MapReduce framework for analyzing next-generation DNA sequencing data. *Genome Research* **20(9)**, 1297–1303, 10.1101/gr.107524.110. Epub 2010 Jul 19. (2010). 10.1101/gr.107524.110 PMC2928508 20644199

[35] Cibulskis, K. *et al.* Sensitive detection of somatic point mutations in impure and heterogeneous cancer samples. *Nature Biotechnology* **31(3)**, 213–219, 10.1038/nbt.2514. Epub 2013 Feb 10. (2013). 10.1038/nbt.2514 PMC3833702 23396013

